# Antimicrobial PAA/PAH Electrospun Fiber Containing Green Synthesized Zinc Oxide Nanoparticles for Wound Healing

**DOI:** 10.3390/ma14112889

**Published:** 2021-05-27

**Authors:** Marina Bandeira, Bor Shin Chee, Rafaele Frassini, Michael Nugent, Marcelo Giovanela, Mariana Roesch-Ely, Janaina da Silva Crespo, Declan M. Devine

**Affiliations:** 1 Materials Research Institute, Athlone Institute of Technology, N37 HD68 Athlone, Ireland; b.schee@research.ait.ie (B.S.C.); mnugent@ait.ie (M.N.); 2 Área do Conhecimento de Ciências Exatas e Engenharias, Universidade de Caxias do Sul, Rua Francisco Getúlio Vargas, 1130, Caxias do Sul 95070-560, RS, Brazil; mgiovan1@ucs.br (M.G.); jscrespo@ucs.br (J.d.S.C.); 3 Instituto de Biotecnologia, Universidade de Caxias do Sul, Rua Francisco Getúlio Vargas, 1130, Caxias do Sul 95070-560, RS, Brazil; rfrassin@ucs.br (R.F.); mrely@ucs.br (M.R.-E.)

**Keywords:** zinc oxide nanoparticles, antimicrobial, electrospinning, polymer fiber, wound healing

## Abstract

Wound infections are the main complication when treating skin wounds. This work reports a novel antimicrobial material using green synthesized zinc oxide nanoparticles (ZnONPs) incorporated in polymeric fibers for wound healing purposes. ZnONPs are a promising antimicrobial nanomaterial with high activity against a range of microorganisms, including drug-resistant bacteria. The electrospun fibers were obtained using polyacrylic acid (PAA) and polyallylamine hydrochloride (PAH) and were loaded with ZnONPs green synthesized from *Ilex paraguariensis* leaves with a spherical shape and ~18 nm diameter size. The fibers were produced using the electrospinning technique and SEM images showed a uniform morphology with a diameter of ~230 nm. EDS analysis proved a consistent dispersion of Zn in the fiber mat, however, particle agglomerates with varying sizes were observed. FTIR spectra confirmed the interaction of PAA carboxylic groups with the amine of PAH molecules. Although ZnONPs presented higher antimicrobial activity against *S. aureus* than *E. coli*, resazurin viability assay revealed that the PAA/PAH/ZnONPs composite successfully inhibited both bacteria strains growth. Photomicrographs support these results where bacteria clusters were observed only in the control samples. The PAA/PAH/ZnONPs composite developed presents antimicrobial activity and mimics the extracellular matrix morphology of skin tissue, showing potential for wound healing treatments.

## 1. Introduction

Skin wounds are a common condition caused by burns, surgery, skin diseases, traumas, among others, causing physical and psychological stress to the patient. Thus, the development of biomaterials to improve wound closure has been addressed [1,2]. The addition of antimicrobial agents to these biomaterials is also essential, as infections are reported to be the main reason of wound complications [3,4].

Zinc oxide nanoparticles (ZnONPs) are a multifunctional material due to a range of intrinsic properties, such as semiconductivity, UV radiation absorption, low cytotoxicity, and antimicrobial activity against a range of microorganisms [5,6]. In addition, this nanomaterial has shown antimicrobial activity against drug-resistant bacteria strains and is considered to be a potential coadjutant in therapy [7]. In the biomedical field, ZnONPs are applied in drug delivery systems, cancer, and tissue regeneration therapies, among others [8,9].

Literature has reported the role of ZnONPs in tissue regeneration [10], where it has been successfully employed for wound healing [11,12]. For instance, Gong et al. [13] and Khalid et al. [14] observed wound healing improvement when applying a polymer material loaded with ZnONPs into the wounds. Both studies showed not only the antimicrobial efficacy of ZnONPs but also its importance for faster tissue regeneration and wound closure.

Electrospinning is a low-cost and facile approach to obtain micro and nanofibrous material that is comparable to the extracellular matrices of skin tissue. For instance, scaffolds obtained using this technique have three-dimensional structures formed by thin fibers and presents porosity, flexibility, and mechanical resistance [15]. Several works report the advantages of electrospun fibers for wound healing, such as cell adhesion and proliferation, flexibility, breathability, and facility for drug release [15,16,17].

Different approaches have been used to incorporate zinc oxide into electrospun fibers varying the polymer or the incorporation of particles. In general, electrospun polymer/zinc oxide fibers were obtained by mixing the particles in the polymer solution before the electrospinning, by adding a zinc precursor in the polymer solution and exposing the electrospun fiber to a calcination process, by incorporating the particles after obtaining the fibers, and by performing an in situ synthesis in the electrospun mat [18,19]. In the field of wound healing, electrospun gelatin/ZnONPs were successfully applied as wound dressing [20,21]. Chitosan, polyvinyl alcohol, and sodium alginate are also examples of polymers used to prepare electrospun composites with ZnONPs for tissue engineering [22,23].

Polyacrylic acid (PAA) and polyallylamine hydrochloride (PAH) are weak polyelectrolytes that present negative and positive charges when in solution, respectively. These materials form polymeric complexes due to their opposite charges and their biocompatibility has been reported for a range of applications, like tissue engineering, drug delivery, and implant coatings [24,25,26]. These polyelectrolytes are water-soluble and require no chemical crosslinking agent as the amino groups of PAH react with the carboxyl groups of the PAA molecules, providing moisture stability and mechanical resistance [25].

The ZnONPs were synthesized using *Ilex paraguariensis* leaves via a sustainable process, which is beneficial not only regarding environmental preservation but also to obtain a product with promising low cytotoxicity and side effects, as no hazardous material is used in the process [27,28]. Literature reports the synthesis of ZnONPs using different plants [29,30,31,32,33,34]. For example, ZnONPs obtained with *Aloe vera* extract and *Stevia* leaves showed enhanced antibacterial effect against different pathogens [30,34]. Another study used *Hibiscus subdarifa* leaves and achieved ZnONPs with antidiabetic properties [31]. Here, ZnONPs were biosynthesized using *Ilex paraguariensis* (IP) leaves extract. This plant is found in South America and is reported to have high concentrations of active compounds, such as chlorogenic acid and caffeine, which are essential for the green synthesis of metal and metal oxide nanoparticles [35,36,37].

In this work, we report a novel antimicrobial material for wound healing applications by combining the antimicrobial activity of green synthesized ZnONPs with a biocompatible electrospun PAA/PAH polyelectrolyte fiber mat, that resembles the extracellular matrix morphology of the skin tissue.

## 2. Materials and Methods

### 2.1. Materials

Materials used to synthesize the fibers include polyacrylic acid 25% *w*/*v* solution (PAA, MW ~345,000, (CH_2_CH(CO_2_H)-)n, CAS n. 9003-01-4, Polysciences, Warrington, PA, USA) and polyallylamine hydrochloride (PAH, MW ~17,500, (CH_2_CH(CH_2_NH_2_·HCl))n, CAS n. 71550-12-4, Sigma Aldrich, St. Louis, MO, USA). The zinc oxide nanoparticles were produced via green synthesis using zinc nitrate hexahydrate (Zn(NO_3_)_2_·6H_2_O, CAS n. 0196-18-6, Sigma-Aldrich), zinc acetate dihydrate ((CH_3_COO)_2_·2H_2_O, CAS n. 5970-45-6, Sigma-Aldrich), and ethanol 99% (C_2_H_5_OH, CAS n. P1000510153081, Didática Artigos para Laboratório Ltd.a.). The plant (*Ilex paraguariensis*) leaves were collected in the municipality of Caxias do Sul, RS, Brazil, and identified at the Natural Science Museum of University of Caxias do Sul (registration n° 46.334).

The antimicrobial activity was performed using bacterial strains of *Staphylococcus aureus* ATCC 25923 (LGC Standards, Middlesex, UK) and *Escherichia coli* ATCC 35218 (LGC Standards, Middlesex, UK). Bacteria cells were cultured using Mueller Hinton broth (MHB, LAB114-A, Lab M Limited, Lancashire, UK) and resazurin sodium salt (C_12_H_6_NNaO_4_, CAS n. 62758-13-8, Sigma-Aldrich) was used to evaluate bacteria viability. Glutaraldehyde 50% *w*/*v* solution (C_5_H_8_O_2_, CAS n. 111-30-8, Alfa Aesar, Ward Hill, MA, USA), phosphate buffer solution (PBS, MDL n. MFCD00131855, Sigma Aldrich), and ethanol 99% were used to prepare bacteria samples for SEM analysis.

### 2.2. Methods

#### 2.2.1. Green Synthesis of ZnONPs

The green synthesized ZnONPs were prepared as described in a previous work [28]. Briefly, to obtain the plant extract, 100 g L^−1^ of IP leaves, previously cleaned, dried (1.5 h at 60 °C) and ground in a mill, were heated at 50 °C and stirred during 20 min with two different solutions: pure distilled water and a 50% (*v*/*v*) ethanol/water solution (ethanolic extract). The extract was then filtrated and centrifuged for 20 min at 5000 rpm to remove suspended particles. For the green synthesis, a zinc precursor (zinc nitrate or zinc acetate) in a Zn(II) concentration of 21.8 g L^−1^ was added in 50 mL of the IP extract solution previously prepared. The solution was stirred for 1 h at room temperature and then heated at 70 °C for 4 h without stirring. At the end of this procedure, the mixture was thermally treated in a hot air oven at 140 °C for 1 h and then calcinated at 400 °C for 1 h. The final powder was collected for further characterization.

#### 2.2.2. Preparation of PAA/PAH/ZnO Electrospun Fibers

**Figure 1:**
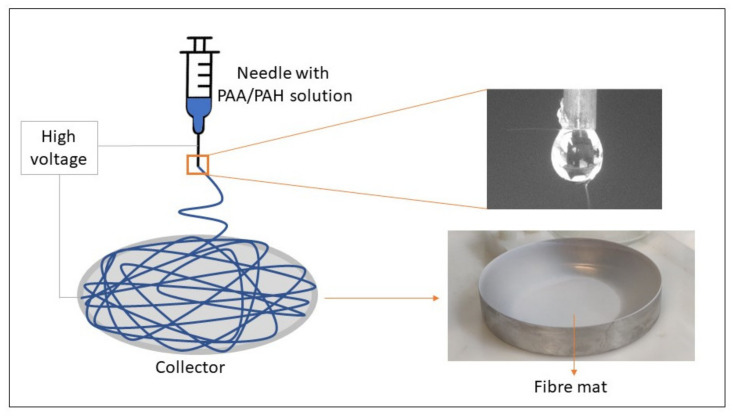
Production of electrospun PAA/PAH fibers.

A polymer solution was prepared by first mixing 1.6 mL of PAA with 0.4 mL of ethanol/water 40% *v*/*v*. Then, 0.11 g of PAH was added to the solution and mixed until homogeneous. The PAA/PAH solution was electrospun using a 0.9 needle and an electrical potential of 10 kV. A fixed working distance from the tip of the needle to the stainless-steel plate of 10 cm was used and the pressure was kept between 0.015 to 0.020 bar. A scheme of the electrospinning process is illustrated in Figure 1. The methodology was adapted from Boas et al. [25] and based on preliminary studies (data not shown).

The fiber mat was then collected from the stainless-steel plate and annealed at 140 °C for 6 h for polymer crosslinking. After cooling to room temperature, the electrospun polymer blend was immersed in 20 mL of a ZnONPs aqueous solution (1 g L^−1^) for 40 min to allow the absorption of nanoparticles on the fiber mat. The fiber mat was then removed from the solution and dried for 2 h at 37 °C. The ZnONPs solution was prepared by sonicating the solution for 15 min following a 10 min of magnetic stirring.

#### 2.2.3. Field Emission Scanning Electron Microscopy (FESEM) Coupled with Energy Dispersive Spectroscopy (EDS)

The morphology of the fiber was examined with a field emission scanning electron microscope (FESEM, TESCAN, model MIRA 3, Brno, Czech Republic) equipped with an energy beam of 10 kV. Prior to the analysis, the samples were placed in an aluminum stub and were covered with a thin layer of gold by a sputtering method (Denton Vacuum, Desk V, Moorestown, NJ, USA) for 30 s at 0.13 mbar vacuum to perform the analysis. EDS analysis of Zn was performed coupled with FESEM using a silicon drift detector (SDD). The mean diameter of the fibers was determined using the Image J (version 1.51j8) software [38], considering the measurements of 15 fibers within a single field of view.

#### 2.2.4. Zinc Quantification by Inductively Coupled Plasma-Optical Emission Spectrometry (ICP-OES)

The amount of ZnONPs absorbed in the fibers was quantified on a single sample by means of zinc concentration using ICP-OES (Thermo Scientific 7200-duo, Waltham, MA, USA), according to Standard Methods EPA 3050b (1996) [39].

#### 2.2.5. Fourier Transform Infrared Spectroscopy (FTIR)

The interaction of the electrospun PAA and PAH polymers was addressed by FTIR spectroscopy (Perkin Elmer, Waltham, MA, USA). Cast films of pure PAA and PLA were also analyzed for comparison. The spectra were registered in a range of 4000 to 650 cm^−1^ and a resolution of 1 cm^−1^ was employed.

#### 2.2.6. Antimicrobial Assay of ZnONPs

*Staphylococcus aureus* ATCC 25923 and *Escherichia coli* ATCC 25922 were cultured and grown in an exponential phase in MHB medium at 37 °C. The viability of bacterial cells when exposed to different concentrations of ZnONPs and to the electrospun fiber mats was analyzed in a 96-well plate using the resazurin cell viability assay. Resazurin indicates cell viability by changing from a blue to a pink color upon chemical reduction resulting from aerobic respiration due to cell growth. Thus, the reduction of the dye is proportional to the viable cells present in the solution and the minimum inhibitory concentration is determined when no dye reduction occurs.

Overnight cultures of the two types of bacteria were diluted to a concentration of approximately 1.0 × 10^4^ colony-forming units per milliliter (CFU mL^−1^). An autoclaved aqueous suspension of ZnONPs (10 mg mL^−1^) was diluted in MHB in different concentrations (from 15 to 100 µg mL^−1^). Then, 100 µL of different ZnONPs suspension and 5 µL of the diluted bacteria were added to each well. Two controls without nanoparticles were also included in each plate: a positive control (100 μL of MHB with 5 µL of the diluted bacteria) and a negative control (only 100 μL of MHB). After overnight incubation at 37 °C, 15 µL of resazurin (0.15 mg mL^−1^) was added to each well and mixed thoroughly for 2 h, and plates were then subjected to absorbance measurement at 600 nm. The test was performed in two individual triplicates.

#### 2.2.7. Antimicrobial Assay of Electrospun Fibers

A similar protocol was followed to evaluate the antimicrobial activity of the fiber mats, where 0.5 cm^2^ of PAA/PAH/ZnONPs and PAA/PAH fibers were incubated in a 96-well plate with 50 μL of MHB and 2.5 μL of bacteria solution (1×10^4^ CFU mL^−1^) prepared as previously described. After overnight incubation at 37 °C, 7.5 μL of resazurin was added to each well and incubated for a further 2 h. Then, the fibers were removed, and the absorbance was measured at 600 nm. A positive control (50 μL of MHB and 2.5 μL of bacteria solution) and negative control (50 μL of MHB) were included in each plate and the tests were performed in two individual triplicates.

Microbial adhesion to the fibers was evaluated using FESEM analysis. For this, the antimicrobial test was performed as described above, but without the addition of resazurin. Thus, after overnight incubation, the media was removed from the wells and bacteria cells were fixed with 3% glutaraldehyde solution in PBS (*v*/*v*) for 15 min at 4 °C, following a sequence of dehydration with 30, 50, 70, 90, and 100% (*v*/*v*) ethanol aqueous solutions for 10 min. The fiber mats were then kept in a desiccator until the analysis was performed. The samples were gold-coated in sputtering equipment (Baltec model SCD 005, Canonsburg, PA, USA) for 110 s at 0.1 mbar prior to the FESEM analysis in high vacuum mode with a maximum beam voltage of 9 kV and back-scattered electron mode.

## 3. Results and Discussion

### 3.1. ZnONPs Green Synthesized

The ZnONPs were synthesized using *Ilex paraguariensis* leaves extract using a green synthesis approach. The synthesis was evaluated according to the zinc source and plant extract solvent, and the ZnONPs were characterized according to their morphology, size, and cytotoxicity. These results were carefully discussed in a previous work [28]. Briefly, the sample produced with zinc nitrate and ethanolic plant extract resulted in the most uniform particle with a spherical shape and a diameter of 18 ± 5 nm. This sample also presented the least cytotoxicity against L929 mouse fibroblast cells and was chosen to be applied in the electrospun fiber here developed.

### 3.2. Characterization of Electrospun Fibers

**Figure 2:**
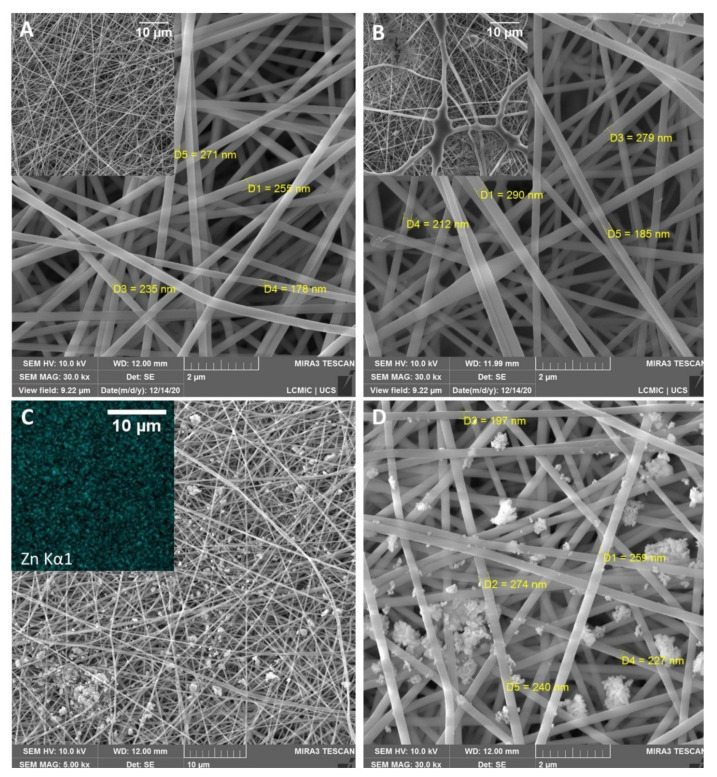
Photomicrograph of (**A**) PAA/PAH fiber mat thermally treated, (**B**) PAA/PAH fiber without thermal treatment, (**C**) PAA/PAH fiber containing ZnONPs and EDS analysis of elemental zinc and, (**D**) Amplification of PAA/PAH fibers containing ZnONPs.

The morphology and size of the polymer electrospun fibers were examined using SEM analysis as shown in Figure 2. The thermal treatment of the fibers (Figure 2A). resulted in an increase in the uniformity of the fibers, resulting in an average diameter of ~230 nm, while the non-thermal treated sample (Figure 2B) presented irregular morphology showing a mix of beads with a diameter up to 4 µm and fibers with a diameter of ~240 nm. PAA and PAH are polyelectrolytes that form electrostatic complexes due to the opposite charges of carboxylate (–COO^−^) and amine (–NH_3_^+^) groups present in the PAA and PAH molecules, respectively [25]. The thermal treatment results in the crosslinking of the polyelectrolytes through these carboxylate and amine groups, which improves the uniformity of the fibers and moisture stability [25]. The morphology of the electrospun scaffold is also found to be suitable for wound dressing as it mimics the extracellular matrix of skin tissue, having a fibrous three-dimensional structure, which provides mechanical support for cell attachment and growth and benefits wound healing [40,41].

Figure 2C. presents the morphology of the fiber after the incorporation of ZnONPs with an elemental analysis of Zn identified by EDS analysis. the incorporation process of ZnONPs did not affect the morphology and size of the fiber mat, as it maintained its characteristics. The green synthesized ZnONPs were characterized in a preview work and have a spherical shape and an average size of 18 nm [28].

However, even though EDS analysis shows a homogeneous dispersion of ZnONPs in the fiber mat, different sizes of agglomerates of nanoparticles were formed. This result might be related to the high concentration of the ZnONPs solution. Considering that it is now known that the fibers can absorb a considerable amount of ZnONPs, more diluted nanoparticle solutions should be used in an attempt to decrease the particle agglomerates. Different studies have produced electrospun fiber containing zinc oxide but usually by incorporating the ZnONPs into the polymer solution, which resulted in a more uniform dispersion of nanoparticles without particle agglomeration [22,23]. Nonetheless, this approach was not possible in this study due to the non-homogeneous solution formed when mixing the polyelectrolytes with the ZnONPs.

The concentration of zinc oxide incorporated in the fibers was measured using ICP-OES. The analysis resulted in a concentration of 9.01 wt % of Zn(II). Considering that ZnO is constituted of 80.3% of Zn(II), the amount of ZnONPs in the fiber mat is 11.2 wt %. The fiber mats presented a thickness of 30 ± 3 µm and a grammage of 0.83 ± 0.20 mg cm^2^. There was no significant difference between the fibers with and without ZnONPs in these aspects. Therefore, the concentration of ZnONPs per area of the fiber mat is approximately 0.09 mg cm^2^.

### 3.3. Fourier Transform Infrared Spectroscopy (FTIR)

**Figure 3:**
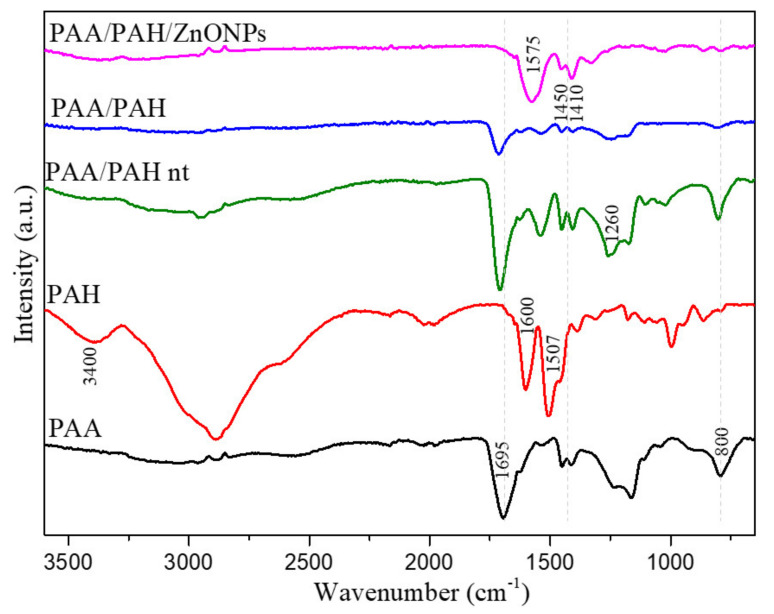
FTIR spectra of fiber mats and PAA and PLA cast films.

FTIR spectra of the different fiber mats in comparison to the cast film of pure PAA and PAH are shown in Figure 3. The main peaks are reported and assigned to their respective groups in Table 1. Peaks 1450 and 1410 cm^−1^ were seen in every sample and are assigned to C-H vibration of the carbon chain of the polymers. PAH shows the characteristic peaks of primary amines (3400 and 1600 cm^−1^) while the PAA presents a strong peak at 1695 cm^−1^ related to the C=O stretching of carboxylic acid [25]. The peak at 1260 cm^−1^ is also related to the carboxylic acid group but it is associated with the C-O stretching and is seen in both PAA and PAA/PAH nt samples. These peaks related to the carboxylic acid groups are also presented in both non-thermal treated and thermally treated PAA/PAH samples. However, it is absent in the PAA/PAH/ZnONPs spectrum. The peaks between 1580 and 1550 cm^−1^ in the fiber samples are related to the amide groups, which are formed by the reaction of the carboxylic acid with the amine groups during polymer cross-linking [42].

### 3.4. Antimicrobial Activity of ZnONPs

**Figure 4:**
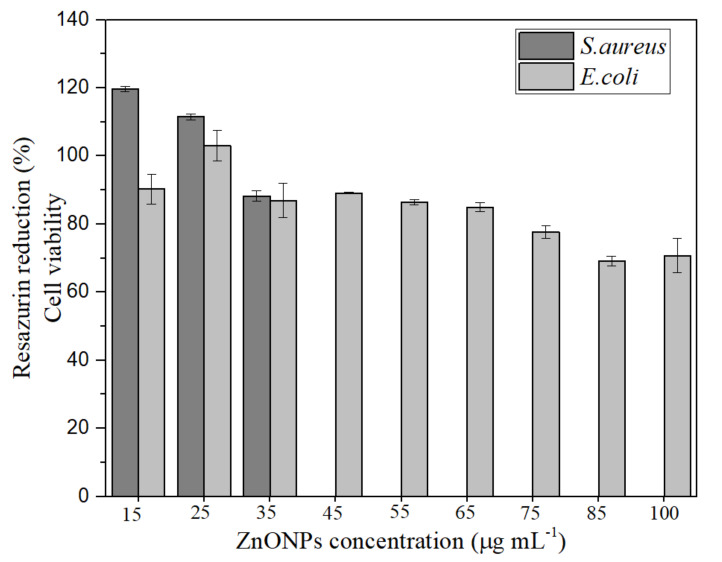
Antimicrobial activity of green synthesized ZnONPs shows higher bacteria inhibition against *S. aureus* strain in comparison to *E. coli* strain.

An antibacterial assay was performed to evaluate the antimicrobial potential of the ZnONPs green synthesized in different concentrations (15 to 55 µg mL^−1^). The results are shown in Figure 4. Overall, ZnONPs showed antimicrobial activity to both bacteria strains. However, a higher antimicrobial activity was observed in Gram-positive (*S. aureus*) compared to Gram-negative (*E. coli*) bacteria. The minimum inhibitory concentration (MIC) is determined as the lower concentration where no resazurin reduction occurred. Therefore, the MIC of ZnONPs for the Gram-positive *S. aureus* was 35 µg mL^−1^, while the Gram-negative *E. coli* showed higher resistance with 70% viability in the highest concentration tested (100 µg mL^−1^).

Previous works have also reported higher toxicity of ZnONPs to Gram-positive bacteria than Gram-negative [43,44,45,46]. This feature is related to the different cell structures of Gram-positive and Gram-negative bacteria. The antimicrobial activity mechanism of metal and metal oxide nanoparticles involves membrane damage, nanoparticle internalization, metal ions, and reactive oxygen species (ROS) that affect cell metabolism [47,48]. For instance, Gram-positive cells have a thicker peptidoglycan layer than Gram-negative bacteria. Nonetheless, Gram-negative have an outer membrane surrounded by lipids and proteins, which can result in higher resistance to nanoparticle and metal ions penetration into the cell. Moreover, Gram-positive bacteria have a more negatively charged surface, which facilitates the attraction of positive ions, such as Zn(II) ions that can be released from the dissolution of ZnONPs [49,50].

### 3.5. Antimicrobial Activity of Electrospun Fibers

**Figure 5:**
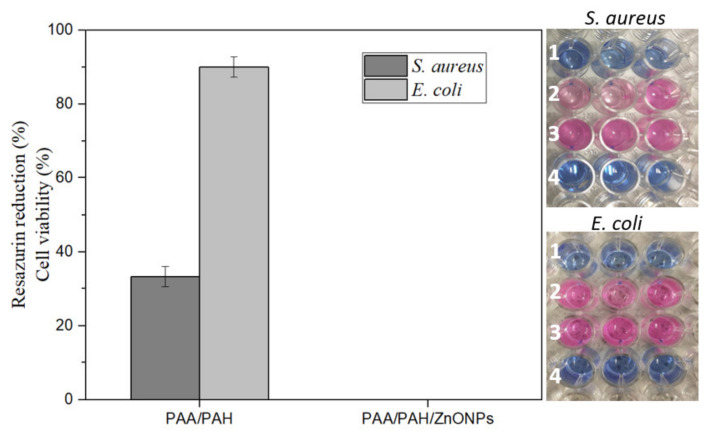
Resazurin antimicrobial assay demonstrates total bacteria growth inhibition for the electrospun fiber containing ZnONPs in comparison to the PAA/PAH fiber mat (1) PAA/PAH/ZnO, (2) PAA/PAH fiber, (3) positive control and, (4) negative control.

The antimicrobial activity of the electrospun fibers is presented in Figure 5. The PAA/PAH fiber decreased the *S. aureus* cell viability to around 40%. Conversely, the *E. coli* showed higher resistance to the fiber mat with only a 10% drop of cell viability in comparison to the positive control. The PAA/PAH/ZnONPs fiber composite was efficient to inhibit both bacteria strains as no dye reduction was observed, confirming the role of ZnONPs in the antimicrobial efficacy of the electrospun fibers. This result can also be observed in the well-plate pictures in Figure 5, where the replicates in row 1 did not reduce the resazurin dye, maintaining the blue color, while the PAA/PAH in row 2 presented some degree of dye reduction in comparison to the row 3, which represents the maximum cell viability. Row 4 showed no dye reduction, as no bacteria were incubated in these wells (negative control).

The final concentration of ZnONPs when testing the PAA/PAH/ZnONPs sample was found to be 900 µg mL^−1^, once a fiber mat of 0.5 cm^2^ (which contains 0.045 mg of ZnONPs) was placed in 0.05 mL of MHB in each well. Therefore, the antimicrobial activity of the PAA/PAH/ZnONPs was expected as it surpassed the MIC for both bacteria strains. Even though the MIC for *E. coli* was not observed up to a concentration of 100 µg mL^−1^, total cell growth inhibition indicates that the MIC was achieved when using the 900 µg mL^−1^ concentration.

**Figure 6:**
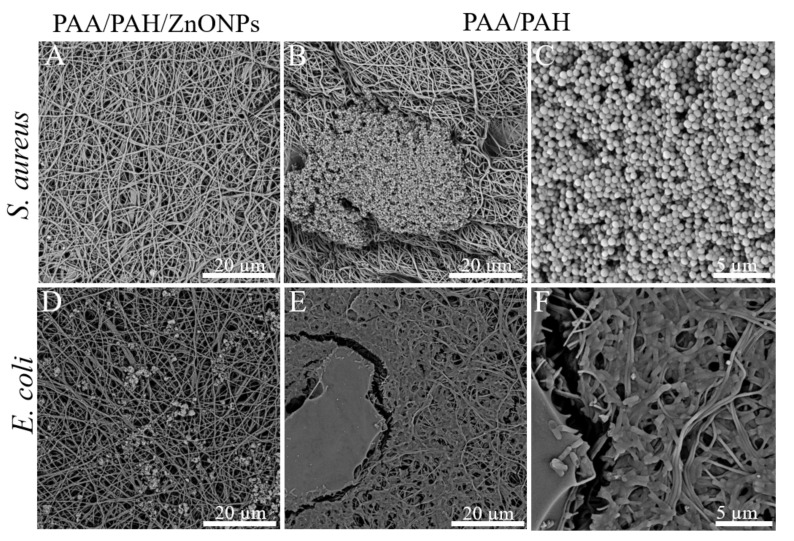
SEM photomicrographs indicate that the presence of ZnONPs inhibits bacteria growth (**A**) PAA/PAH/ZnONPs sample after 24 h incubation with *S. aureus,* (**B**) PAA/PAH sample after 24 h incubation with *S. aureus,* (**C**) amplification of image B to highlight the *S. aureus* bacteria cluster morphology, (**D**) PAA/PAH/ZnONPs sample after 24 h incubation with *E. coli*, (**E**) PAA/PAH sample after 24 h incubation with *E. coli*, and (**F**) amplification of image C to highlight the *E. coli* bacteria morphology and attachment to the fibers.

FESEM photomicrographs of the fiber after performing the antimicrobial assay are presented in Figure 6. This analysis corroborates with the resazurin assay as no bacteria growth was observed in the PAA/PAH/ZnONPs fibers for both *E. coli* and *S. aureus* strains. In addition, *S. aureus* formed isolated colony clusters in the control sample (PAA/PAH) while the *E. coli* covered nearly the whole surface of the scaffold forming a biofilm, confirming the results observed in the resazurin dye assay.

The FESEM photomicrographs also show that *S. aureus* clusters are formed by spherical cells and are presented only on the surface of the fibers (Figure 6B,C). Conversely, *E. coli* cells are rod in shape and are attached to the fibers, using the structure of the material to support their growth, as shown in Figure 6E,F.

## 4. Conclusions

Polymer fibers were successfully prepared using the electrospinning technique and posteriorly loaded with green synthesized ZnONPs with spherical shape and a diameter of ~18 nm. The fiber scaffold was formed by uniform PAA/PAH fibers and presented an even distribution of ZnONPs. However, some agglomerates of the nanomaterials were observed.

The electrospun fibers showed a similar morphology to the extracellular matrix of skin tissue and the addition of ZnONPs inhibited both Gram-negative and Gram-positive bacteria strains. These features display the potential of this novel material to be used for wound dressings aiming for faster wound healing and infection prevention. In future work, the particle agglomeration needs to be improved and the biocompatibility of the material must be addressed to confirm the application for tissue regeneration.

## Figures and Tables

**Table 1:** Description of FTIR peaks.

Wavenumber (cm^−1^)	Group
3400	N-H stretching (amine)
1695	C=O stretching (carboxylic acid)
1600	N-H bending (amine)
1575	-NH bending (amide)
1450, 1410	C-H bending
1260	C-O stretching

## Data Availability

All data generated or analyzed during this study are included in the article.
